# Clinical utilization of artificial intelligence-based COVID-19 pneumonia quantification using chest computed tomography – a multicenter retrospective cohort study in Japan

**DOI:** 10.1186/s12931-023-02530-2

**Published:** 2023-10-05

**Authors:** Hiromu Tanaka, Tomoki Maetani, Shotaro Chubachi, Naoya Tanabe, Yusuke Shiraishi, Takanori Asakura, Ho Namkoong, Takashi Shimada, Shuhei Azekawa, Shiro Otake, Kensuke Nakagawara, Takahiro Fukushima, Mayuko Watase, Hideki Terai, Mamoru Sasaki, Soichiro Ueda, Yukari Kato, Norihiro Harada, Shoji Suzuki, Shuichi Yoshida, Hiroki Tateno, Yoshitake Yamada, Masahiro Jinzaki, Toyohiro Hirai, Yukinori Okada, Ryuji Koike, Makoto Ishii, Naoki Hasegawa, Akinori Kimura, Seiya Imoto, Satoru Miyano, Seishi Ogawa, Takanori Kanai, Koichi Fukunaga

**Affiliations:** 1 Division of Pulmonary Medicine, Department of Internal Medicine, Keio University School of Medicine, 35 Shinanomachi, Shinjuku-ku, Tokyo, 160-8582 Japan; 2 Department of Respiratory Medicine, Graduate School of Medicine, Kyoto University, 54 Kawahara-cho, Shogoin, Sakyo-ku, Kyoto, 606-8507 Japan; 3 Department of Clinical Medicine (Laboratory of Bioregulatory Medicine), Kitasato University School of Pharmacy, Tokyo, Japan; 4 Department of Respiratory Medicine, Kitasato University, Kitasato Institute Hospital, Tokyo, Japan; 5 Department of Infectious Diseases, Keio University School of Medicine, Tokyo, Japan; 6 Department of Respiratory Medicine, JCHO (Japan Community Health care Organization), Saitama Medical Center, Saitama, Japan; 7 Department of Respiratory Medicine, Juntendo University Faculty of Medicine and Graduate School of Medicine, Tokyo, Japan; 8 Department of Pulmonary Medicine, Saitama City Hospital, Saitama, Japan; 9 Department of Radiology, Keio University School of Medicine, Tokyo, Japan; 10 Department of Statistical Genetics, Osaka University Graduate School of Medicine, Suita, Japan; 11 Department of Genome Informatics, Graduate School of Medicine, the University of Tokyo, Tokyo, Japan; 12 Laboratory for Systems Genetics, RIKEN Center for Integrative Medical Sciences, Kanagawa, Japan; 13 Health Science Research and Development Center (HeRD), Tokyo Medical and Dental University, Tokyo, Japan; 14 Department of Respiratory Medicine, Nagoya University Graduate School of Medicine, Nagoya, Japan; 15 Institute of Research, Tokyo Medical and Dental University, Tokyo, Japan; 16 Division of Health Medical Intelligence, Human Genome Center, the Institute of Medical Science, the University of Tokyo, Tokyo, Japan; 17 M&D Data Science Center, Tokyo Medical and Dental University, Tokyo, Japan; 18 Department of Pathology and Tumor Biology, Kyoto University, Kyoto, Japan; 19 Division of Gastroenterology and Hepatology, Department of Internal Medicine, Keio University School of Medicine, Tokyo, Japan

**Keywords:** Artificial intelligence (AI)-based analysis, Computer Vision System, Pneumonia, Post-acute COVID-19 syndrome, SARS-CoV-2 infection

## Abstract

**Background:**

Computed tomography (CT) imaging and artificial intelligence (AI)-based analyses have aided in the diagnosis and prediction of the severity of COVID-19. However, the potential of AI-based CT quantification of pneumonia in assessing patients with COVID-19 has not yet been fully explored. This study aimed to investigate the potential of AI-based CT quantification of COVID-19 pneumonia to predict the critical outcomes and clinical characteristics of patients with residual lung lesions.

**Methods:**

This retrospective cohort study included 1,200 hospitalized patients with COVID-19 from four hospitals. The incidence of critical outcomes (requiring the support of high-flow oxygen or invasive mechanical ventilation or death) and complications during hospitalization (bacterial infection, renal failure, heart failure, thromboembolism, and liver dysfunction) was compared between the groups of pneumonia with high/low-percentage lung lesions, based on AI-based CT quantification. Additionally, 198 patients underwent CT scans 3 months after admission to analyze prognostic factors for residual lung lesions.

**Results:**

The pneumonia group with a high percentage of lung lesions (N = 400) had a higher incidence of critical outcomes and complications during hospitalization than the low percentage group (N = 800). Multivariable analysis demonstrated that AI-based CT quantification of pneumonia was independently associated with critical outcomes (adjusted odds ratio [aOR] 10.5, 95% confidence interval [CI] 5.59–19.7), as well as with oxygen requirement (aOR 6.35, 95% CI 4.60–8.76), IMV requirement (aOR 7.73, 95% CI 2.52–23.7), and mortality rate (aOR 6.46, 95% CI 1.87–22.3). Among patients with follow-up CT scans (N = 198), the multivariable analysis revealed that the pneumonia group with a high percentage of lung lesions on admission (aOR 4.74, 95% CI 2.36–9.52), older age (aOR 2.53, 95% CI 1.16–5.51), female sex (aOR 2.41, 95% CI 1.13–5.11), and medical history of hypertension (aOR 2.22, 95% CI 1.09–4.50) independently predicted persistent residual lung lesions.

**Conclusions:**

AI-based CT quantification of pneumonia provides valuable information beyond qualitative evaluation by physicians, enabling the prediction of critical outcomes and residual lung lesions in patients with COVID-19.

**Supplementary Information:**

The online version contains supplementary material available at 10.1186/s12931-023-02530-2.

## Background

Since the emergence of the first case of coronavirus disease (COVID-19) in 2019, more than 676 million cases have been reported worldwide [1, 2]. Significant efforts have been made to develop vaccines and treatments, such as systemic corticosteroids and remdesivir [3, 4], to improve disease prognosis. However, there have been recurring outbreaks in Japan [5] and may acquire stronger virulence in the future, forcing us to prepare for the next pandemic. Because there are heterogeneous disease courses of COVID-19, early identification of patients at risk of severe disease is important for the appropriate use of medical resources.

Chest computed tomography (CT) is extensively used to diagnose COVID-19 and to predict its severity [6, 7]. In the early years of the COVID-19 pandemic, qualitative assessments of pneumonia by radiologists were useful for predicting disease severity [8–10]; however, the reproducibility of evaluation by radiologists is problematic [11]. In fact, semi-quantitative analysis of pneumonia and well-aerated lung volumes using CT density has shown a better prediction of severity than qualitative assessments by radiologists [12, 13]. Furthermore, artificial intelligence (AI) -based CT analyses have recently become useful in diagnosing and predicting the severity of COVID-19 [14–16]. The advantage of AI-based analysis is that it can quickly, easily, and reproducibly quantify pneumonia without intra- or interobserver variability. However, reports on the usefulness of AI-based CT analysis in predicting COVID-19 severity are limited to a small number of cases at a single center over a short duration [15, 17]. Only a few studies have compared the detailed clinical characteristics or complications during hospitalization of patients based on the volume of pneumonia quantified using AI-based analyses [6].

Since the latter half of 2020, various systemic symptoms have been reported to persist after the acute phase of COVID-19 [18] and to cause long-term lung sequelae [19]. The pathogenesis of the sequelae is not well understood, and the analysis of structural changes using CT may be important for understanding them. Qualitative CT evaluation by radiologists has shown that lung sequelae are frequently accompanied by residual shadows three months after COVID-19 [20]; however, only a few studies have performed AI-based CT quantification of residual lesions [21, 22]. Moreover, no study has examined the clinical characteristics of patients with residual lesions using AI-based CT quantification. As there is no established management strategy to improve lung sequelae, further understanding of the underlying structural changes in relation to the clinical features is warranted.

It was hypothesized that AI-based CT quantification of pneumonia would not only be useful for predicting outcomes in the acute phase but also for the objective assessment of lung sequelae. This study aimed to investigate (1) the usefulness of AI-based CT quantification of COVID-19 pneumonia in predicting critical outcomes using a multicenter database with long-term duration, and (2) the longitudinal change in AI-based CT quantification of residual lesions and the clinical characteristics of patients with persistent pneumonia after the acute phase of infection.

## Methods

### Study population

This retrospective cohort study utilized the clinical data collected by the Japan COVID-19 Task Force, a nationwide multicenter consortium in Japan [23, 24]. Informed or oral consent was obtained from all patients, and the study was approved by the Ethics Committee of Keio University School of Medicine (20,200,061) and related institutions. A flowchart of the study is presented in Figure E1 in the Online Supplement. A total of 1,410 patients with COVID-19 were recruited from four institutions (Keio University Hospital, Juntendo University Hospital, Saitama Medical Center, and Saitama City Hospital) between February 2020 and July 2022. Among these patients, 210 were excluded based on the following criteria: unavailable chest CT images (N = 193) and CT images inappropriate for analysis (N = 17). Therefore, the baseline chest CT analysis included 1,200 patients. Additionally, chest CT scans at 3 months after admission were analyzed in 198 patients, after excluding 1,002 patients based on the following criteria: unavailable for 3-month chest CT (N = 993) and CT images inappropriate for analysis (N = 9).

### Clinical data

The following clinical data were collected from patients with COVID-19: demographics, medical history, clinical signs and symptoms, laboratory test data, radiographic observations, need for oxygen support, treatment, complications, and outcomes. Laboratory and radiographic data were collected within 48 h of the initial visit or admission. Complications were collected as they arose during hospitalization. The attending physicians at each facility reviewed and evaluated the chest CT images for qualitative pneumonia. The critical outcomes included [25, 26] conditions requiring the support of a high-flow oxygen device, invasive mechanical ventilation (IMV), or death, corresponding to an ordinal scale of 6–8 in the ACTT-1 clinical trial [4].

### CT acquisition

All the CT images were obtained after full inspiration. Images of the entire lung with a slice thickness of 1–5 mm were reconstructed using standard kernels. The CT scanners used were the SOMATOM series (Siemens Healthineers), Aquilion series (Canon Medical Systems), Revolution series (GE Healthcare), Discovery series (GE Healthcare), and BrightSpeed (GE Healthcare).

### AI-Based image analysis

**Fig. 1:**
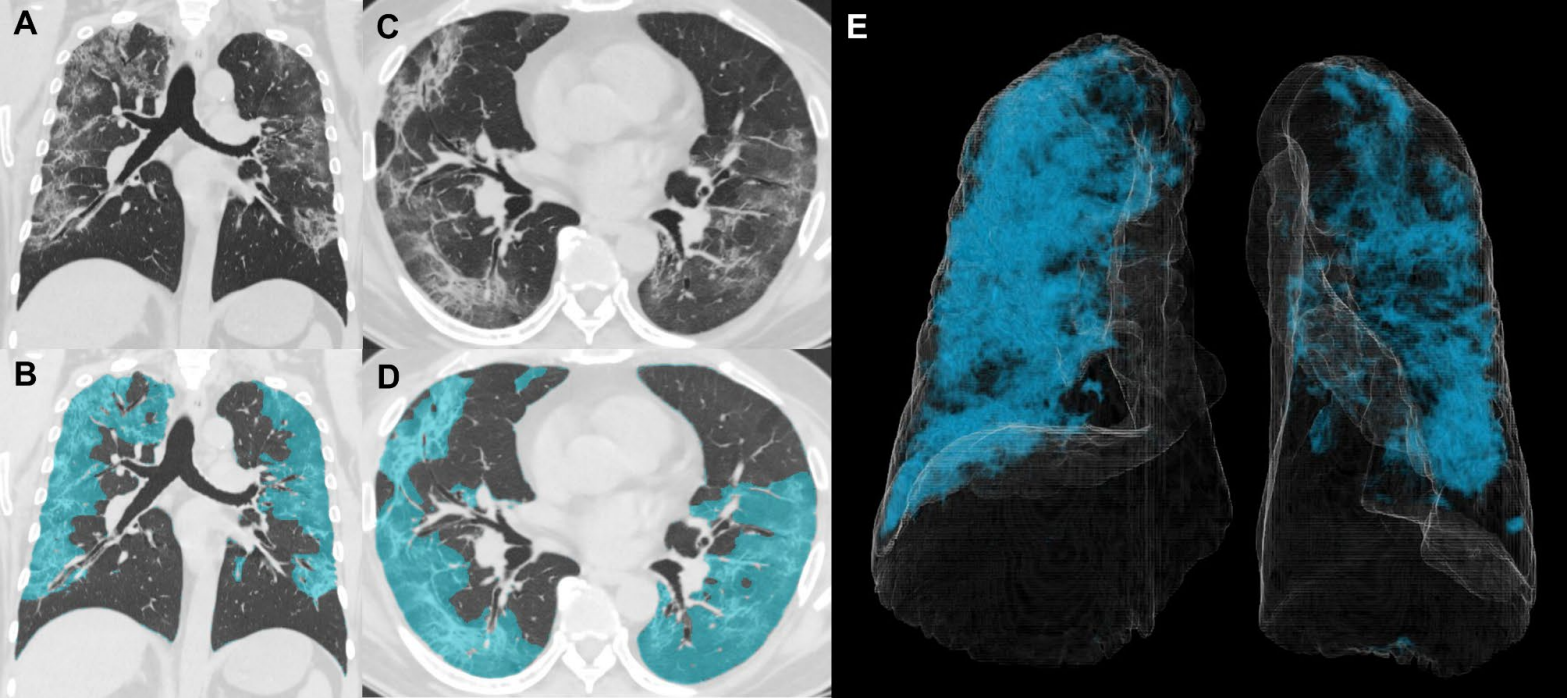
Image of one patient with COVID-19 showing segmentation of the entire lung and pneumonia. (**A-D**) Chest CT images of severe COVID-19 cases (**A**: axial, **C**: coronal) and segmentation of pneumonia lesions (highlighted in Blue) by AI-based analysis (**B**: axial, **D**: coronal). (**E**) Segmentation of the total lung (White curved surface) and pneumonia (Blue highlighted) using 3D viewer images. In this representative case, the total lung volume was 3890 mL, the volume of lung lesions was 1314 mL, and the percentage of lung lesions was 33.8%

Segmentation of pneumonia and the total lung was performed using SYNAPSE VINCENT software (FUJIFILM, Tokyo, Japan). This software was developed to quantify abnormal CT patterns in idiopathic pulmonary fibrosis, and we measured the volume of these abnormal CT patterns as pneumonia shadows in patients with COVID-19. The abnormal CT patterns were automatically segmented by the pre-trained lung disease segmentation model, which classified each voxel in the lungs into nine parenchymal patterns: normal lungs, bronchi, vessels, reticulation, ground-glass opacities (GGO), honeycombing, consolidation, hyperlucency, and nodules. The architecture of the lung disease segmentation is a simple 2D convolutional neural network consisting of six consecutive convolutional layers and two pooling layers. The input is a fixed 32 × 32 pixels image. The output is a single pixel that predicts the class probabilities of the center pixel in the input image. This network was trained with 304 HRCT scans from patients with diffuse lung diseases with manually annotated ground truth labels. Therefore, the model was able to learn local features such as the Hounsfield unit (HU) value distributions and texture patterns for each parenchymal pattern. The three patterns of GGO, consolidation, and reticulation of the nine parenchymal patterns were quantified as abnormal CT patterns in SYNAPSE VINCENT software since these were relevant image findings of COVID-19. The details of the lung disease segmentation model have been described previously [27]. Figure 1 A-E demonstrates an example of the segmentation of pneumonia lesions and whole lungs from one included patient (total lung volume; 3890 mL, volume of lung lesions; 1314 mL, percentage of lung lesions [% lung lesions]; 33.8%). The percentage of lung lesions at admission and residual lesions (% residual lesions) 3 months after admission, were defined as volume divided by total lung volume.

### Statistical analysis

The clinical characteristics of the two groups were compared by dividing the included patients into three tertiles, with the top 1/3 defined as pneumonia with a high percentage of lung lesions and the bottom 2/3 as pneumonia with a low percentage of lung lesions on CT images. The same split into three tertiles was performed for the percentage of residual lesions on the 3-month CT scan. Continuous variables were compared using unpaired t-tests or Mann–Whitney U tests, depending on the normality of distribution, and categorical variables were compared using the chi-square test. To evaluate the relationship between quantitative pneumonia and each outcome, a multivariable logistic regression analysis was performed, adjusted for qualitative evaluation of pneumonia by physicians, days from onset to admission, and previously reported factors, such as age, sex, body mass index (BMI), smoking history, hypertension, diabetes, cardiovascular disease, and chronic kidney disease (CKD) [25, 28, 29]. We also used the area under the receiver operating characteristic (ROC) curve (AUC) to predict critical outcomes, and the cut-off was determined by the Youden index. To evaluate the association with quantitative residual lung lesions, a multivariable analysis was conducted adjusted for the variables that were indicated as factors associated with residual lung lesions in the univariate logistic regression analysis. Finally, we predicted % residual lesions on the 3-month CT scan based on % lung lesions on admission, using linear regression analysis. All statistical analyses were performed using JMP 16 software (SAS Institute Japan Ltd., Tokyo, Japan). Visualization was performed using the R Bioconductor package ggalluvial.

## Results

### Comparison of clinical features between the groups with high and low percent lung lesions on CT images

**Fig. 2:**
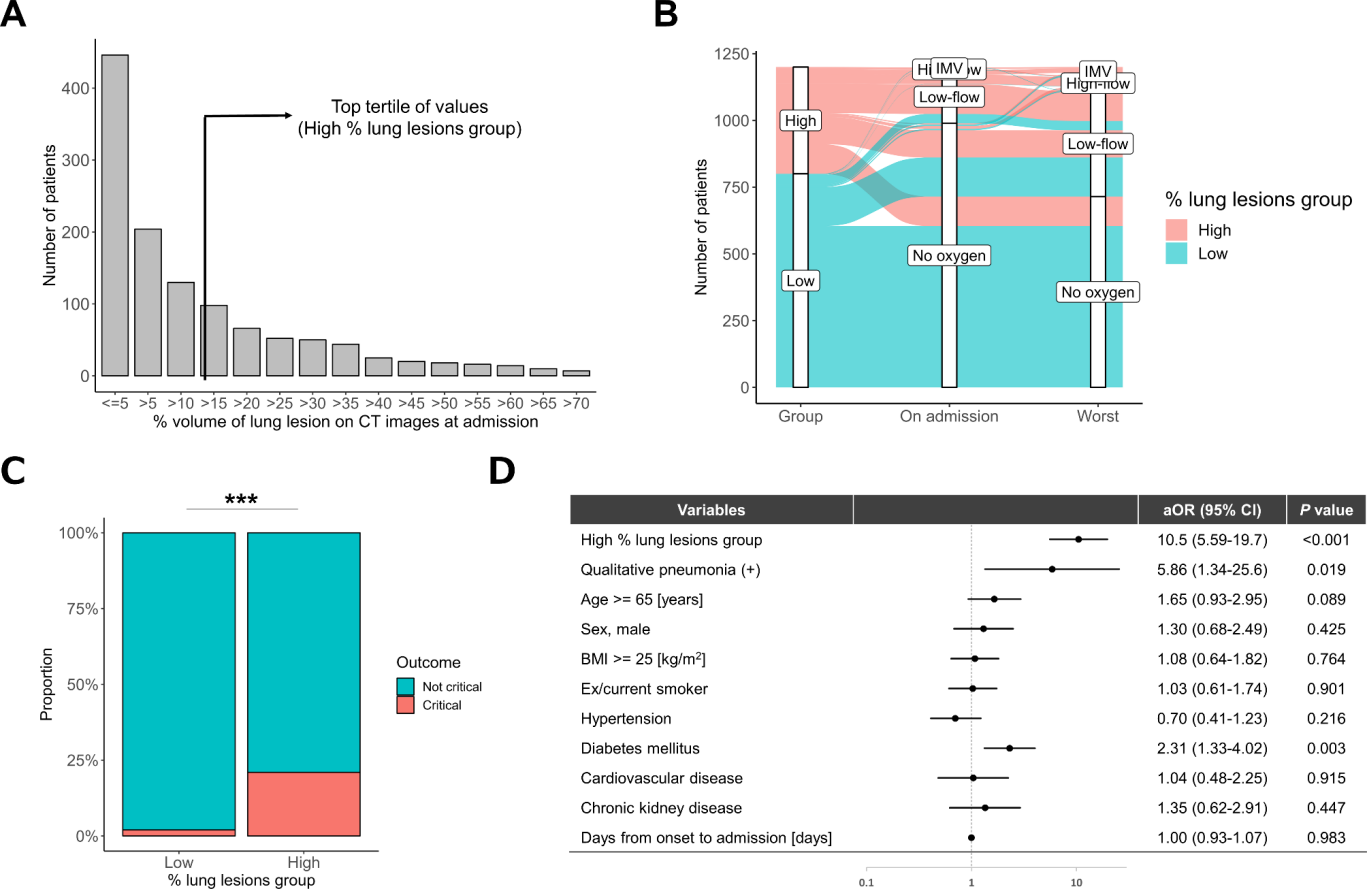
Relationship between the volume of pneumonia on admission and disease severity in hospitalized patients with COVID-19. (**A**) Distribution of percentage volume of pneumonia and definition of pneumonia with high % lung lesions group (top tertile of values). (**B**) Alluvial diagram comparing oxygen demand at admission and at worst during the disease course between the pneumonia groups with high and low % lung lesions. (**C**) Comparison of the incidence of critical pneumonia between the pneumonia groups with high and low % lung lesions. (**D**) Forest plot showing multivariable logistic regression analysis to evaluate the relationship between critical outcomes and the pneumonia group with high % lung lesions. aOR, adjusted odds ratio; BMI, body mass index; CI, confidence interval; IMV, invasive mechanical ventilation. ***; *P* < 0.001

The distribution of pneumonia cases on admission is shown in Fig. 2A. The clinical characteristics of the pneumonia group with high % lung lesions (≥ 16.0%, N = 400) and the pneumonia group with low % lung lesions (< 16.0%, N = 800) were compared. The high % lung lesions group comprised older individuals, more males, individuals with a higher BMI, and had a higher incidence of hypertension, diabetes, and CKD than did the low % lung lesions group (Table 1). The high % lung lesions group also exhibited higher percentages of systemic symptoms (fever and fatigue) and respiratory symptoms (cough and shortness of breath), elevated levels of inflammatory biomarkers (white blood cell count, C-reactive protein [CRP], and ferritin), and a higher frequency of antiviral and immunosuppressive treatment for COVID-19 than did the low % lung lesions group (Table E1).

**Table 1:** Comparison of the backgrounds of patients with COVID-19 between two groups of pneumonia with high/low percentage of lung lesions on CT images at admission

Parameters		Pneumonia with low % lung lesions group (N = 800)	Pneumonia with high % lung lesions group (N = 400)	*P* value
Age [years]		54.3 (± 17.0)	57.6 (± 16.1)	0.001
Sex, male		527 (65.9)	308 (77.0)	< 0.001
BMI [kg/m^2^]		24.5 (± 6.43)	25.7 (± 5.01)	0.002
Smoking history				0.850
Never		416 (53.8)	210 (54.4)	
Previously or currently		357 (46.2)	176 (45.6)	
Medical history				
Hypertension		230 (28.8)	147 (36.8)	0.005
Diabetes mellitus		125 (15.7)	94 (23.6)	< 0.001
Cardiovascular disease		70 (8.8)	46 (11.6)	0.127
Malignancy		76 (9.6)	36 (9.0)	0.749
Autoimmune disease		58 (7.3)	21 (5.3)	0.184
COPD		25 (3.1)	9 (2.3)	0.398
Asthma		68 (8.6)	18 (4.6)	0.012
Hyperuricemia		88 (11.1)	47 (11.8)	0.704
Chronic liver disease		26 (3.3)	11 (2.8)	0.634
Chronic kidney disease		55 (6.9)	42 (10.6)	0.032
Admission				
Days from onset to admission [days]		4.71 (± 3.00)	6.79 (± 3.73)	< 0.001
Data are as N (%) or mean (standard deviation). Abbreviations: BMI, body mass index; COPD, chronic obstructive pulmonary disease; COVID-19, coronavirus disease.				

### Comparison of clinical outcomes between the groups with high and low percent lung lesions on CT images

The pneumonia group with high % lung lesions not only had a higher percentage of patients requiring high-flow oxygen or IMV support upon admission but also the worst during hospitalization when compared to that of the pneumonia group with low % lung lesions (Fig. 2B). This trend was consistent when stratified by oxygen demand at admission (Figure E2A-B in the Online Supplement). The high % lung lesions group exhibited a higher incidence of critical outcomes than the low % lung lesions group (21.0% vs. 2.0%, *P* < 0.001) (Fig. 2C), and % volume of lung lesions on admission was also higher in critical cases than in noncritical cases (35.3% vs. 12.9%, *P* < 0.001) (Figure E3A in the Online Supplement). In the ROC curve, the % volume of lung lesions on admission predicted a critical outcomes with an AUC of 0.845, a sensitivity of 82.0%, and a specificity of 74.2%, using a cut-off of 17.6 (Figure E3B in the Online Supplement). Patients qualitatively diagnosed with pneumonia by clinicians had higher rates of critical outcomes and conditions requiring oxygen and IMV support than those without pneumonia (Figure E4 in the Online Supplement). In a multivariable analysis adjusted for qualitative evaluation of pneumonia by physicians and previously reported factors, AI-based CT quantification of COVID-19 pneumonia was independently associated with critical outcomes (adjusted odds ratio [aOR] 10.5, 95% confidence interval [CI] 5.59–19.7) (Fig. 2D). Additionally, AI-based CT quantification of pneumonia predicted oxygen requirement (aOR 6.35, 95% CI 4.60–8.76), IMV requirement (aOR 7.73, 95% CI 2.52–23.7), and mortality rate (aOR 6.46, 95% CI 1.87–22.3) (Figure E5 in the Online Supplement). Stratification by epidemic wave demonstrated that the high % lung lesions group had a higher incidence of critical outcomes than did the low % lung lesions group during all epidemic waves in Japan (1st − 3rd waves, 19.5% vs. 2.1%, *P* < 0.001; 4th wave, 25.8% vs. 3.4%, *P* < 0.001; 5th wave, 24.8% vs. 2.3%, *P* < 0.001; 6th − 7th waves, 14.1% vs. 0.0%, *P* < 0.001) (Figure E6 in the Online Supplement).

### Comparison of complications during hospitalization between the groups with high and low percent lung lesions on CT images

**Fig. 3:**
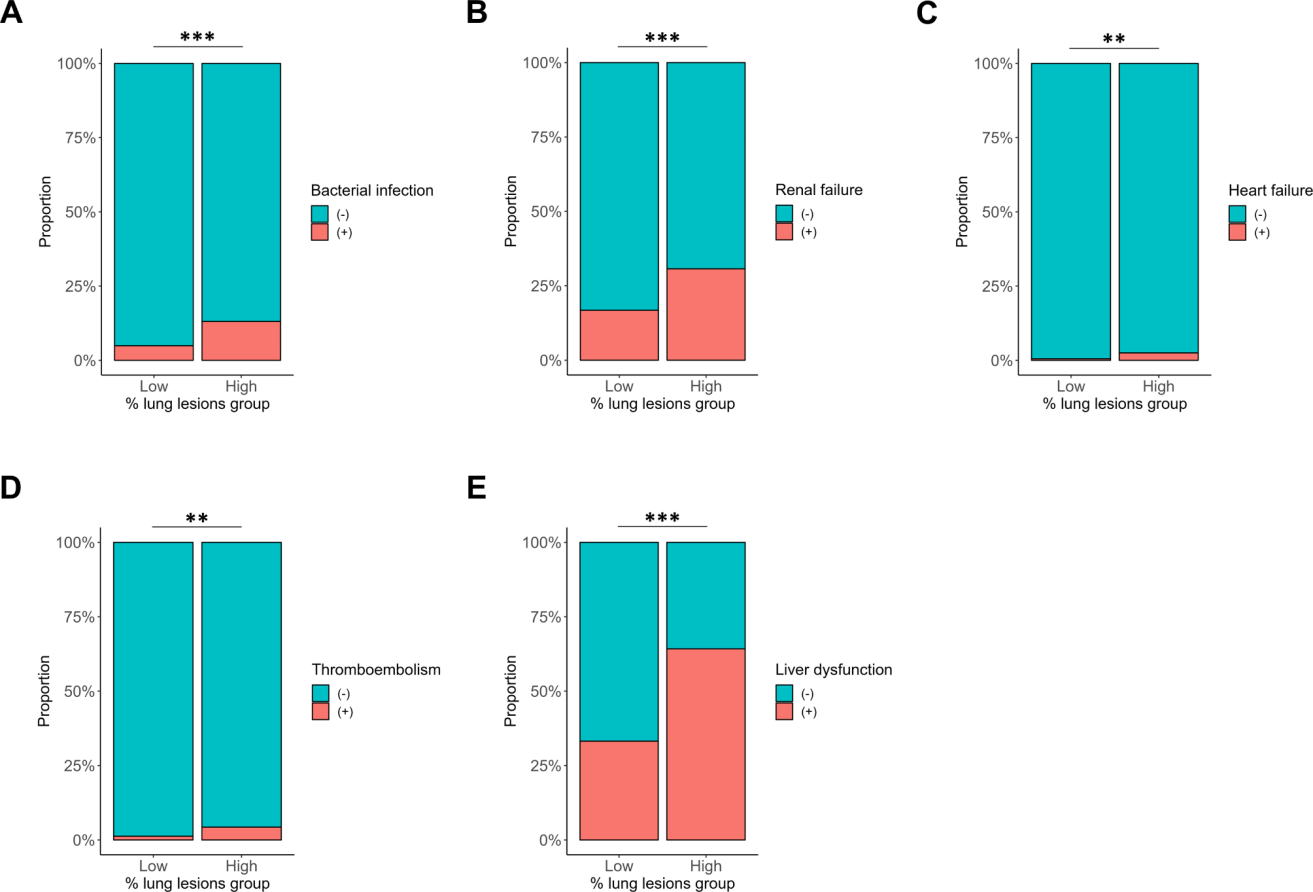
Comparison of the incidence of complications during hospitalization between the pneumonia groups with high and low % lung lesions. (**A**) Bacterial infection, (**B**) renal failure, (**C**) heart failure, (**D**) thromboembolism, and (**E**) liver dysfunction. **; *P* < 0.01, ***; *P* < 0.001

Figure 3 compares the incidence of complications during hospitalization between the pneumonia groups with high and low % lung lesions. The high % lung lesions group exhibited a higher incidence of bacterial infection (13.1% vs. 4.9%, *P* < 0.001), renal failure (30.7% vs. 16.8%, *P* < 0.001), heart failure (2.5% vs. 0.5%, *P* = 0.003), thromboembolism (4.3% vs. 1.3%, *P* = 0.001), and liver dysfunction (64.2% vs. 33.2%, *P* < 0.001) than did the low % lung lesions group.

### Longitudinal change of AI-Based CT quantification over 3 months, and clinical characteristics of patients with residual lung lesions

**Fig. 4:**
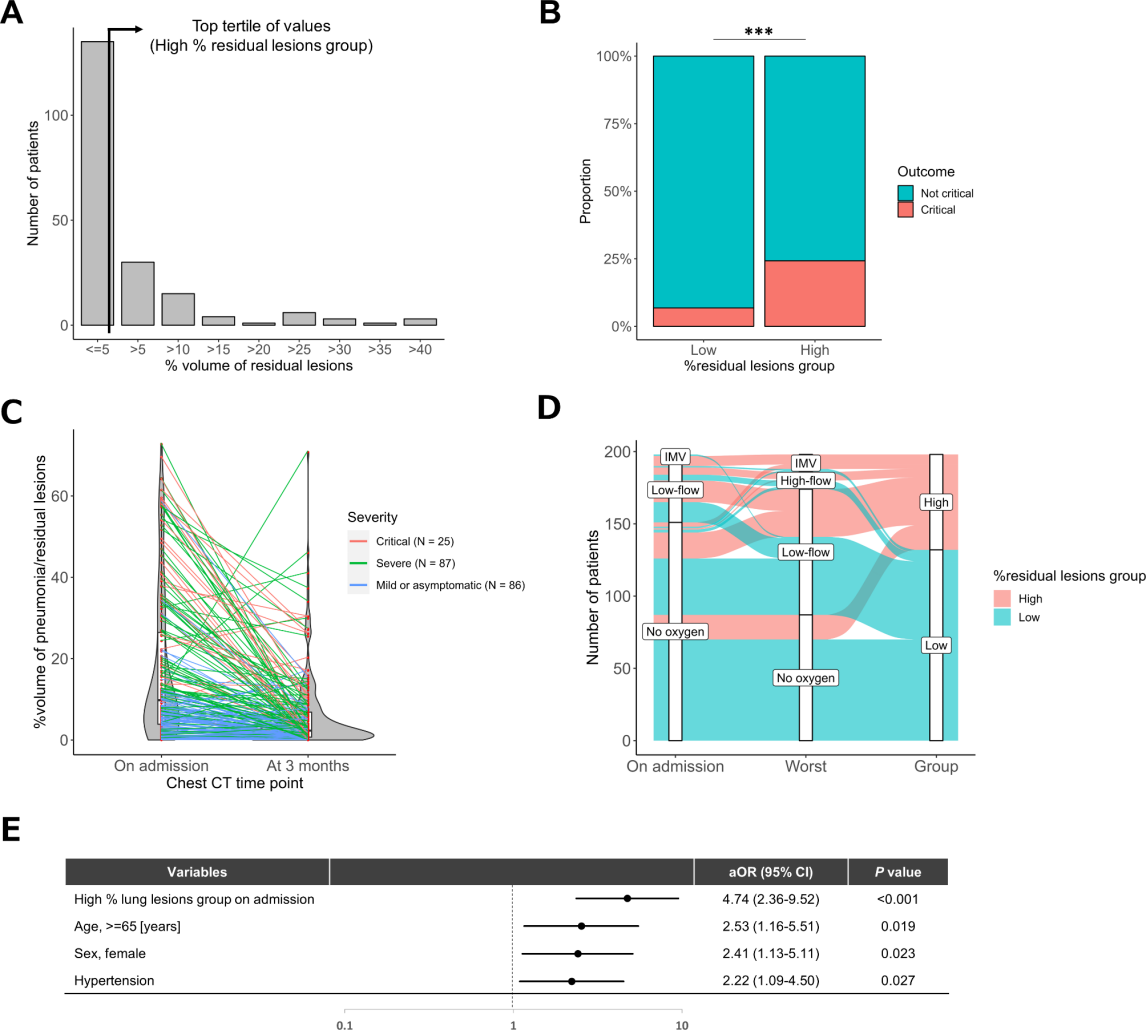
Relationship between volume of residual lung lesions 3 months after admission and disease severity in hospitalized patients with COVID-19. (**A**) Distribution of percentage volume of residual lung lesions and definition of high % residual lesion group (top tertile of values). (**B**) Comparison of the incidence of critical outcomes between the high and low % residual lesion groups. (**C**) Paired comparison of % lung lesions on CT images at admission and % residual lesions among patients with each severity: critical (red lines, N = 25), severe (green lines, N = 87), and mild or asymptomatic (blue lines, N = 86). (**D**) Alluvial diagram comparing oxygen demand at admission and at worst during the disease course between the high and low % residual lesion groups. (**E**) Forest plot showing multivariable logistic regression analysis to evaluate the relationship between each variable and the high % residual lesion group aOR, adjusted odds ratio; CI, confidence interval. ***; *P* < 0.001

A total of 198 patients underwent CT analysis three months following admission. Table E2 compares the clinical characteristics of patients who could and could not undergo CT analysis at three months. The distribution of the percentage of residual lung lesions (% residual lesions) at three months is presented in Fig. 4A. Clinical characteristics were compared between the high %residual lesion group (≥ 4.65%, N = 66) and the low % residual lesion group (< 4.65%, N = 132). Table E3 compares the clinical characteristics, complications, and outcomes of the two groups. The high % residual lesion group comprised older individuals, more female patients, and had higher rates of hypertension, renal failure complications, and critical outcomes (24.2% vs. 6.8%, *P* < 0.001) than the low % residual lesion group (Fig. 4B). Sputum symptoms were significantly more frequent in the high % residual lesion group. Additionally, this group exhibited high neutrophil and low lymphocyte levels, low albumin levels, and elevated levels of lactate dehydrogenase, Krebs von den Lungen-6, D-dimer, and CRP. Regarding outcomes, the group with high % residual lesion also showed a significantly higher intensive care unit admission rate (24.2% vs. 10.7%, *P* = 0.012) and IMV support rate (15.2% vs. 1.5%, *P* < 0.001) than the low % residual lesion group (Table E3). Figure 4 C illustrates the percentage of lung lesions stratified by illness severity from 0 to 3 months. In most cases, the percentage of lung lesions decreased over time; however, patients with a high percentage of residual lesions at three months were more likely to be critical or severe. Using linear regression analysis, we predicted the % volume of residual lesions on 3 months using the values of % volume of lung lesions on admission (r^2^ = 0.11, y = 0.171x + 2.85, *P* < 0.001) (Figure E7 in the Online Supplement). The alluvial chart demonstrated that patients in the top 1/3 of the residual lesions at 3 months were more likely to have required oxygen support upon admission and were more likely to have developed oxygen demand during the course of the disease (Fig. 4D). Next, the predictors of a high percentage of residual lesions at three months were evaluated using univariate analysis (Table E4). Based on these results, the multivariable analysis revealed that in the pneumonia group with high % lung lesions on CT images at admission (aOR 4.74, 95% CI 2.36–9.52), older age (aOR 2.53, 95% CI 1.16–5.51), female sex (aOR 2.41, 95% CI 1.13–5.11), and medical history of hypertension (aOR 2.22, 95% CI 1.09–4.50) were independent predictors of a high percentage of residual lesions at 3 months after admission (Fig. 4E).

## Discussion

This is the first study to investigate the usefulness of AI-based CT quantification of COVID-19 pneumonia for predicting critical outcomes and complications of COVID-19 and to evaluate the longitudinal change in quantification using a large retrospective cohort database. Pneumonia cases with a high percentage of lung lesion classified using AI-based CT quantification was strongly associated with critical outcomes adjusted for other known predictors, including the presence of pneumonia in qualitative assessments and other complications during hospitalization. In addition, longitudinal follow-up of the quantification revealed a high percentage of residual lesions at three months in more cases with critical outcomes. These results show that AI-based CT quantification is a valuable tool that can sufficiently predict the severity of COVID-19 pneumonia and that this tool highlights populations associated with COVID-19 sequelae by assessing persistent pneumonia.

The strength of this study is the use of a novel AI-based CT quantification of pneumonia in a large, multicenter, and long-term cohort. Many previous studies have shown that AI-based CT quantification can predict disease severity in a small number of patients [15, 17]. The large sample size in this study allowed for multivariable analysis and showed that AI-based CT quantification predicts critical outcomes independently of many prognostic factors. In routine clinical practice, especially during a pandemic, it is difficult to perform CT imaging under the same conditions. Although this study included multiple CT scanner models and imaging conditions, it was clinically significant in predicting disease severity. Multiple waves of the COVID-19 pandemic have been confirmed worldwide, including in Japan [30]. Because the characteristics of the viral strain and various other factors, such as the development of therapeutic drugs and vaccines, are involved, the clinical characteristics of patients differ depending on the epidemic period. In this study, we investigated the stratification of epidemic waves and observed that AI-based CT quantification was useful for predicting the severity of COVID-19 regardless of the epidemic waves. This suggests a clinically significant application in the event of future epidemics.

The advantage of AI-based CT analysis is that it enables a more reproducible, quick, and quantitative evaluation of pneumonia than qualitative evaluation by radiologists, and achieves faster analysis than semi-quantitative analysis. The accuracy and promptness of AI-based CT quantification are useful tools for identifying patients with early exacerbation of COVID-19 in clinical practice, although there are several infected patients during the pandemic. In this study, AI-based CT quantification of pneumonia volume was predictive of various clinical outcomes independent of the clinician’s visual assessment of pneumonia. This finding extends previous studies showing that AI-based CT quantification analysis is superior to density-based qualitative analysis for predicting disease severity [31, 32]. Previous reports about pneumonia quantification indicated that the thresholds of % volume of pneumonia were 6.0-8.2% for requiring oxygen support, 23% for requiring IMV support, and 36–40% for mortality, respectively [12, 33–36]. Therefore, we considered the threshold of 16.0% in this study to be an appropriate cut-off value for predicting critical outcomes. Although a large-scale study has proposed severity prediction models using lung CT analysis, multiple clinical data, and radiomics analysis [6, 37], we believe that our simple AI-based CT examination of pneumonia volume (pneumonia with % lung lesions on CT images) can provide prognostic information with similar accuracy and should be more clinically relevant in terms of routine clinical applications.

The pneumonia group with a high % lung lesions showed a higher incidence of multiorgan complications in this study. This is consistent with previous findings of poor outcomes after hospitalization, including bacterial infection [38], renal failure [39], heart failure [40], thrombosis [41], and liver failure [42]. In this context, our data are important as they suggest that AI-based measurement of pneumonia volume can predict complications during hospitalization and allow for early intervention in selected high-risk patients.

Factors related to COVID-19 severity have been proposed to account for the diversity in the natural course of COVID-19 [25, 28, 29]. These previous findings are consistent with our findings that poor prognostic factors, including BMI, diabetes, and CKD, were more frequently observed in patients with a high percentage of pneumonia. Moreover, our data reaffirm previous findings that inflammatory biomarkers, including CRP, procalcitonin, and ferritin, are associated with severe disease [43] and pneumonia [17, 44] because COVID-19 causes a cytokine storm and systemic inflammation with severe disease [45].

COVID-19 causes persistent sequelae, referred to as Long-COVID [46]. A review of CT qualitative evaluations by radiologists showed that lung lesions remained in 70–94% of patients with COVID-19 at 3 months [20, 47–49]. Furthermore, patients with COVID-19 who had residual lung lesions 3 months after COVID-19 onset had poor pulmonary function and severely decreased oxygen saturation during the 6-minute walk test [48]. However, no study has examined pulmonary sequelae using an AI-based CT quantification analysis. In this study, despite the overall improvement in pneumonia volume 3 months after onset, many patients showed residual lesions and more residual lesions were associated with older age, female sex, history of hypertension, and higher severity of COVID-19. These findings are consistent with those of previous reports (a review of radiologists’ qualitative assessments), in which cases of observed residual lesions were associated with older age [48], length of hospitalization, and the need for IMV support [50]. In the present study, we predicted the % volume of residual lesions using the % volume of lung lesions on CT images at admission. AI-based CT quantification analysis at the time of onset is more useful than qualitative analysis for predicting the severity of COVID-19 [31, 32] and may also prevail over the long-term disease course. Longitudinal AI-based CT quantification analysis may be a predictor of long-COVID symptoms, and future studies are warranted to clarify its association with these symptoms.

This study has some limitations. First, the number of cases evaluated 3 months after onset was small owing to drop out. This study used follow-up CT scans obtained 3 months after onset based on previous reports showing that 3 months should be reasonable to evaluate residual lesions, [51] and show a correlation between residual lesions at 3 months and physiological sequelae, including dyspnea [47, 48]. In our study, the severity of the clinical condition on admission was greater in the patients who underwent follow-up CT than in those who did not. This may be a cause of selection bias that the severe patients were more likely to be included in the analysis after 3 months. Second, we did not evaluate the extrapulmonary organs using CT in this study. Several organ abnormalities outside the lungs, including muscle, fat, and coronary artery calcification, are associated with COVID-19 [52–54]. Combining these analyses of extrapulmonary organs with pneumonia volume may lead to improved accuracy in predicting disease severity, and future studies are warranted.

## Conclusions

AI-based CT quantification analysis of pneumonia is promising for providing reproducible information for predicting critical outcomes in patients with COVID-19 and gaining a deeper understanding of residual lung lesions potentially associated with long-term sequelae after the acute stage of COVID-19.

### Electronic supplementary material

Below is the link to the electronic supplementary material.

Supplementary Material 1

## Data Availability

The datasets used and/or analyzed during the current study are available from the corresponding author on reasonable request.

## List of abbreviations

AI: artificial intelligence
aOR: adjusted odds ratio
BMI: body mass index
CI: confidence interval
CKD: chronic kidney disease, COVID-19, coronavirus disease
CRP: C-reactive protein
CT: computed tomography
IMV: invasive mechanical ventilation
