# Supplementary material for: Clinical utilization of artificial intelligence-based COVID-19 pneumonia quantification using chest computed tomography – a multicenter retrospective cohort study in Japan
Source: Respir Res. 2023 Oct 5;24:241. doi: 10.1186/s12931-023-02530-2 (PMC10552312; doi:10.1186/s12931-023-02530-2)
Supplement: Supplementary file 1 — Supplementary Material 1 [file 12931_2023_2530_MOESM1_ESM.docx]

**Clinical Utilization of Artificial Intelligence-Based COVID-19 Pneumonia Quantification**

**Using Chest Computed Tomography – A Multicenter Retrospective Cohort Study in Japan**

**Respiratory Research**

**Online Supplement**

Hiromu Tanaka,^1,*^ Tomoki Maetani,^2,*^ Shotaro Chubachi,^1,†,‡^ Naoya Tanabe,^2,†^ Yusuke Shiraishi,^2^ Takanori Asakura,^1,3,4^ Ho Namkoong,^5^ Takashi Shimada,^1^ Shuhei Azekawa,^1^ Shiro Otake,^1^ Kensuke Nakagawara,^1^ Takahiro Fukushima,^1^ Mayuko Watase,^1^ Hideki Terai,^1^ Mamoru Sasaki,^6^ Soichiro Ueda,^6^ Yukari Kato,^7^ Norihiro Harada,^7^ Shoji Suzuki,^8^ Shuichi Yoshida,^8^ Hiroki Tateno,^8^ Yoshitake Yamada,^9^ Masahiro Jinzaki,^9^ Toyohiro Hirai,^2^ Yukinori Okada,^10,11,12^ Ryuji Koike,^13^ Makoto Ishii,^1,14^ Naoki Hasegawa,^5^ Akinori Kimura,^15^ Seiya Imoto,^16^ Satoru Miyano,^17^ Seishi Ogawa,^18^ Takanori Kanai,^19^ Koichi Fukunaga^1^

^1^ Division of Pulmonary Medicine, Department of Internal Medicine, Keio University School of Medicine, Tokyo, Japan

^2^ Department of Respiratory Medicine, Graduate School of Medicine, Kyoto University, Kyoto, Japan

^3^ Department of Clinical Medicine (Laboratory of Bioregulatory Medicine), Kitasato University School of Pharmacy, Tokyo, Japan,

^4^ Department of Respiratory Medicine, Kitasato University, Kitasato Institute Hospital, Tokyo, Japan,

^5^ Department of Infectious Diseases, Keio University School of Medicine, Tokyo, Japan

^6^ Internal Medicine, JCHO (Japan Community Health care Organization) Saitama Medical Center, Saitama, Japan

^7^ Department of Respiratory Medicine, Juntendo University Faculty of Medicine and Graduate School of Medicine, Tokyo, Japan

^8^ Department of Respiratory Medicine, Saitama City Hospital, Saitama, Japan

^9^ Department of Radiology, Keio University School of Medicine, Tokyo, Japan

^10^ Department of Statistical Genetics, Osaka University Graduate School of Medicine, Suita, Japan

^11^ Department of Genome Informatics, Graduate School of Medicine, the University of Tokyo, Tokyo, Japan

^12^ Laboratory for Systems Genetics, RIKEN Center for Integrative Medical Sciences, Kanagawa, Japan

^13^ Health Science Research and Development Center (HeRD), Tokyo Medical and Dental University, Tokyo, Japan

^14^ Department of Respiratory Medicine, Nagoya University Graduate School of Medicine, Nagoya, Japan

^15^ Institute of Research, Tokyo Medical and Dental University, Tokyo, Japan

^16^ Division of Health Medical Intelligence, Human Genome Center, the Institute of Medical Science, the University of Tokyo, Tokyo, Japan

^17^ M&D Data Science Center, Tokyo Medical and Dental University, Tokyo, Japan

^18^ Department of Pathology and Tumor Biology, Kyoto University, Kyoto, Japan

^19^ Division of Gastroenterology and Hepatology, Department of Internal Medicine, Keio University School of Medicine, Tokyo, Japan

^*^ These authors contributed equally as co-first author.

^†^ These authors contributed equally.

^‡^Corresponding Authors:

Shotaro Chubachi, MD, PhD, Division of Pulmonary Medicine, Department of Internal Medicine, Keio University School of Medicine, 35 Shinanomachi, Shinjuku-ku, Tokyo 160-8582, Japan

Email: [bachibachi472000@z6.keio.jp](mailto:bachibachi472000@z6.keio.jp)

Telephone: +81-3-3353-1211

Naoya Tanabe, MD, PhD, Department of Respiratory Medicine, Graduate School of Medicine, Kyoto University, 54 Kawahara-cho, Shogoin, Sakyo-ku, Kyoto, 606-8507, Japan.

Email: ntana@kuhp.kyoto-u.ac.jp

Telephone: +81-75-751-3830

These two corresponding authors contributed equally.


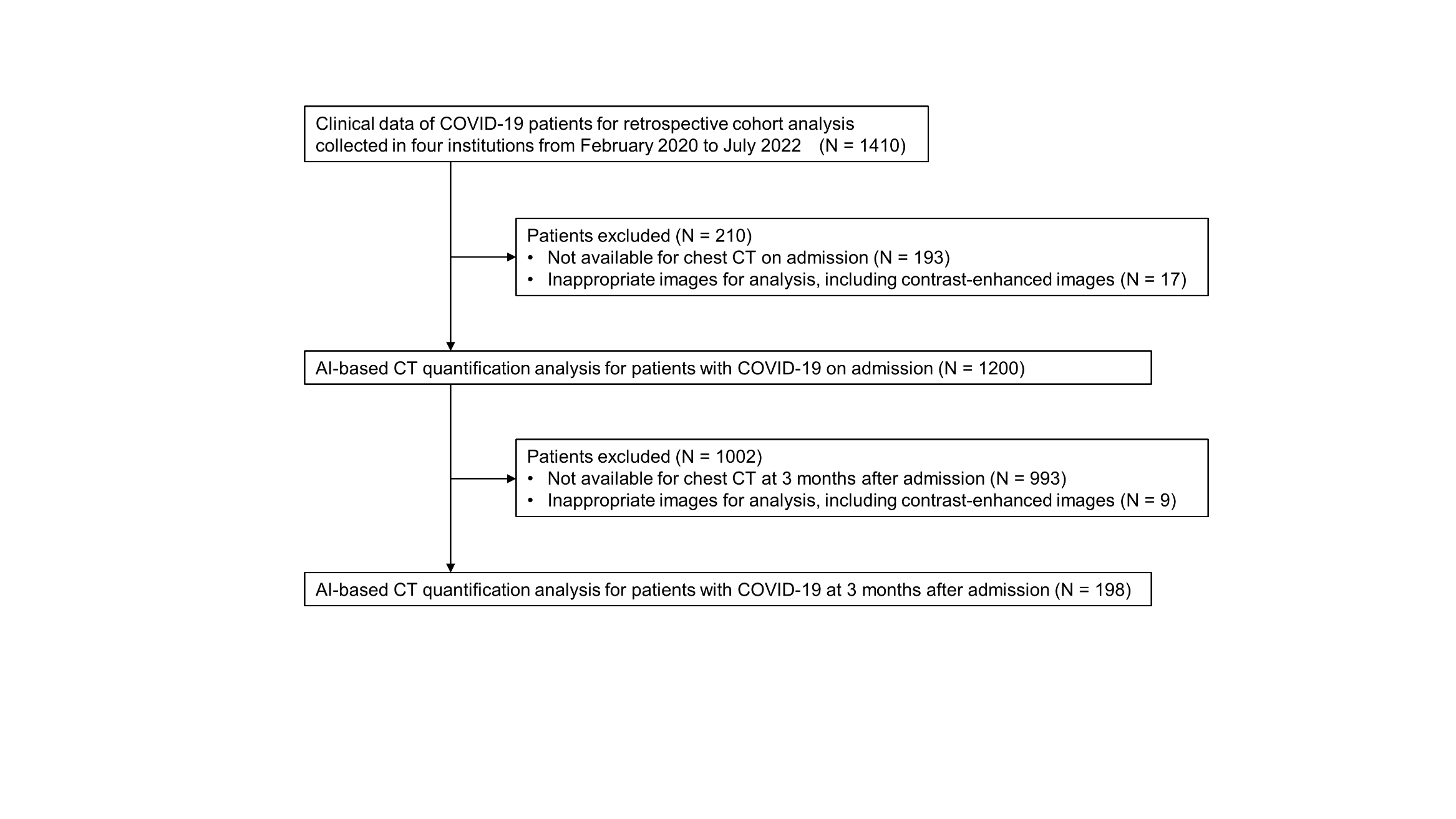


**Figure E1** Consort diagram of patient inclusion. COVID-19; coronavirus disease 2019.

**
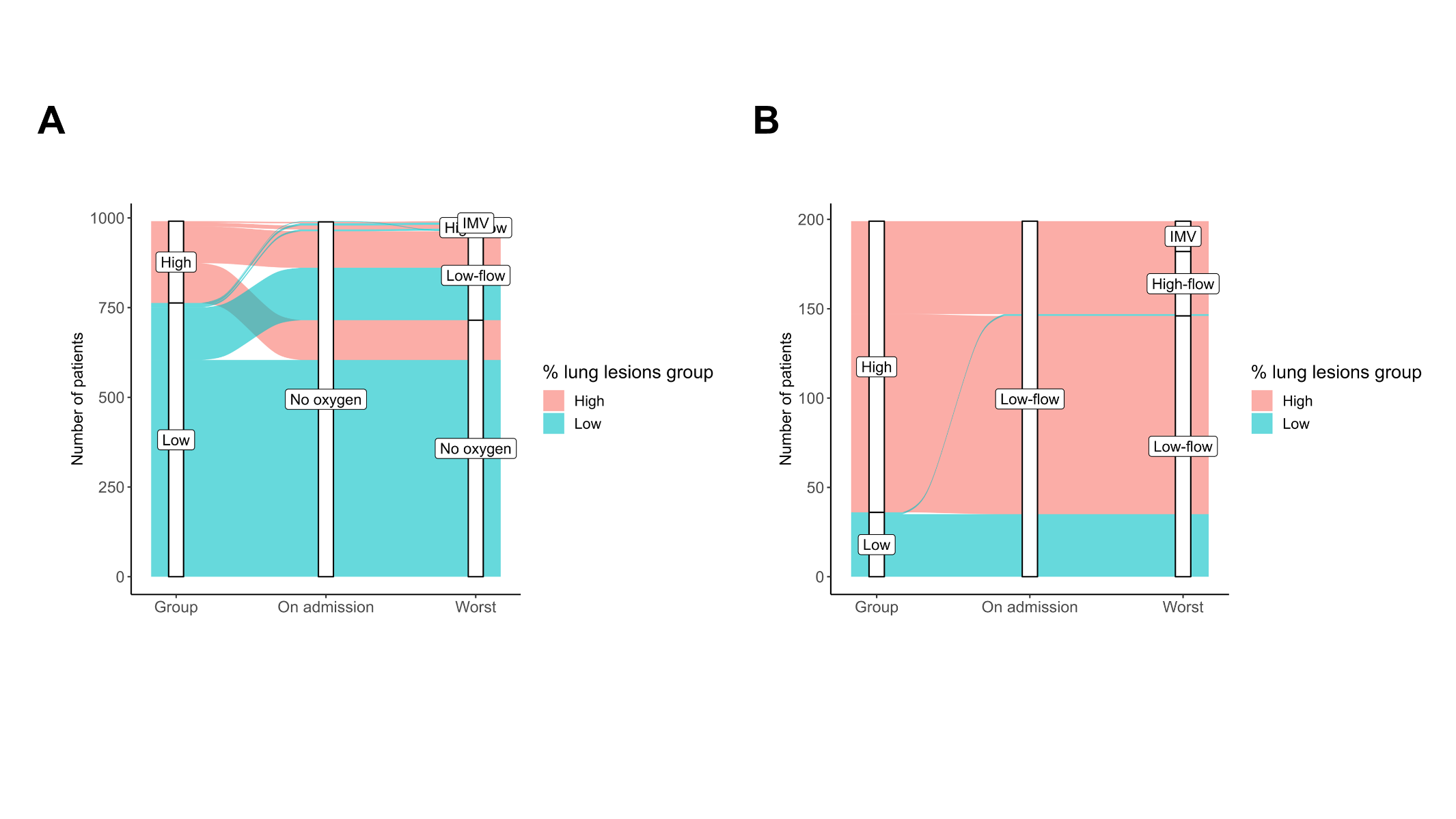
**

**Figure E2** Alluvial diagram comparing oxygen demand at worst during disease course between the groups of pneumonia of high/low % lung lesions on CT images, adjusted by oxygen demand on admission. (A) Comparison in population requiring no oxygen support on admission. (B) Comparison in population requiring low-flow oxygen support on admission. IMV; invasive mechanical ventilation.


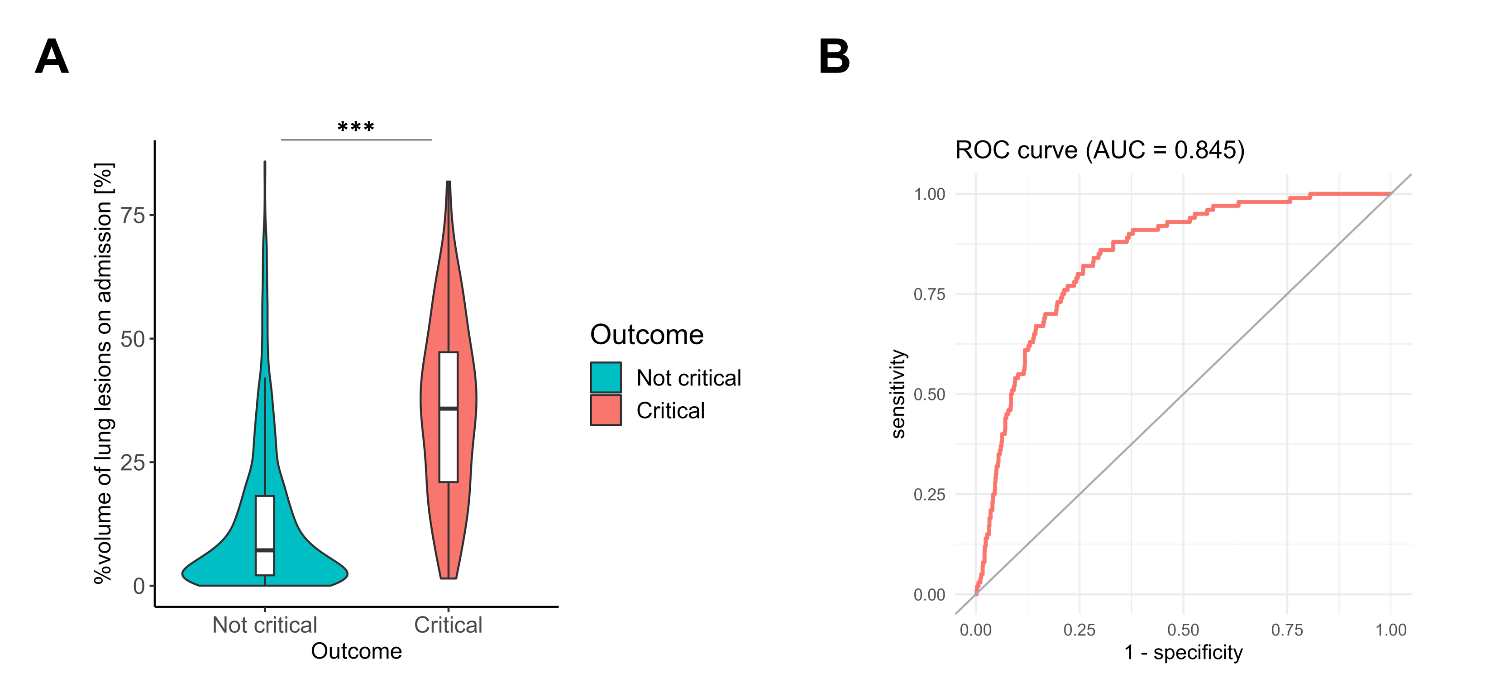


**Figure E3** Prediction of critical outcomes based on percent volume of lung lesions on CT images at admission. (A) The comparison of % lung lesions on admission between critical and not critical cases. (B) ROC curve for prediction of critical outcomes using % lung lesions on admission. ***; *P* < 0.001. AUC; area under the ROC curve, ROC; receiver operating characteristic.


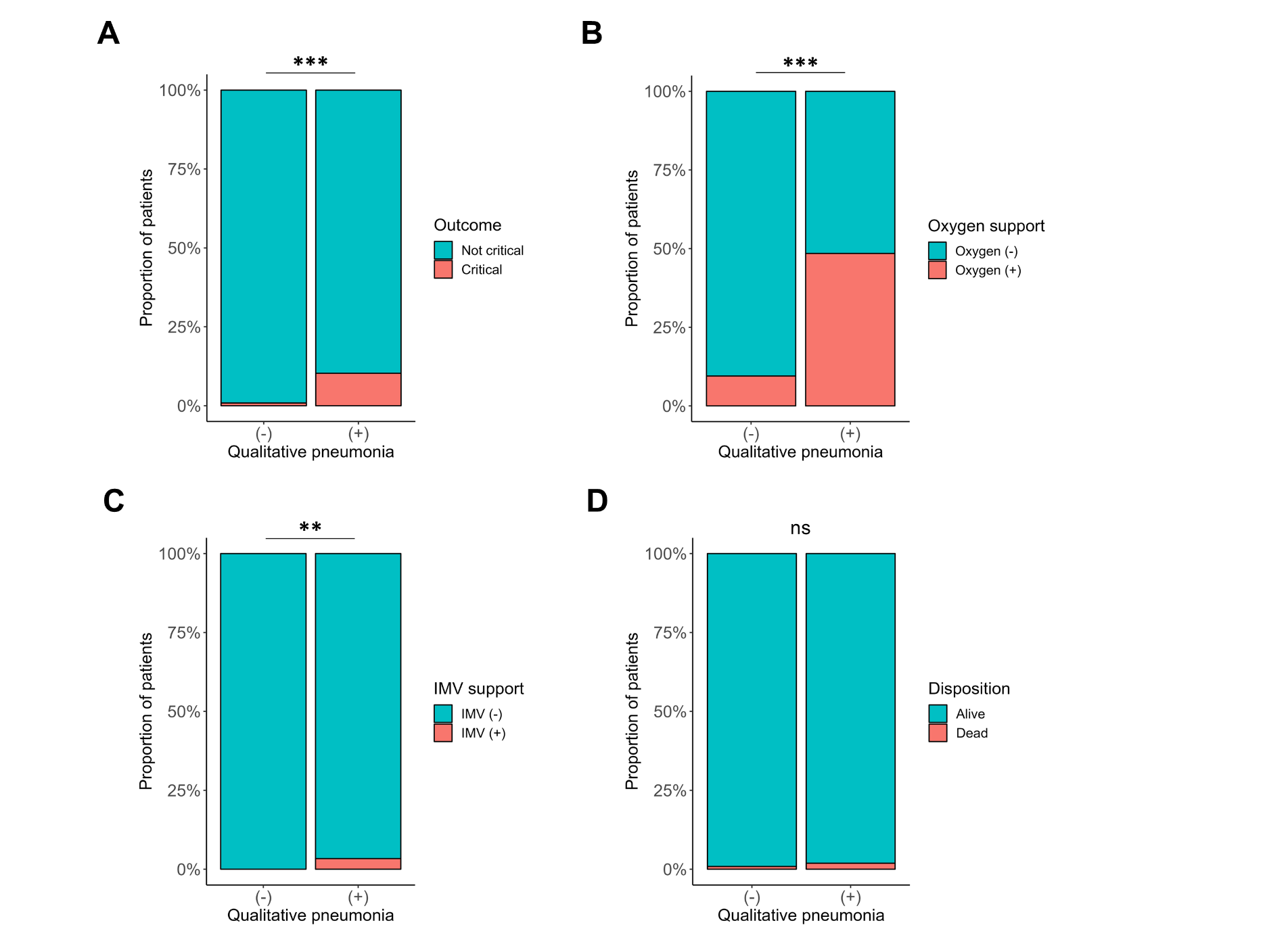


**Figure E4** Comparison of proportion of each outcome between two groups, absence (-) and presence (+) of qualitative pneumonia diagnosed by clinicians. (A) critical outcomes, (B) need for oxygen support during disease course, (C) need for IMV support during disease course, (D) disposition. **; *P* < 0.01, ***; *P* < 0.001. IMV; invasive mechanical ventilation, ns; not significant.


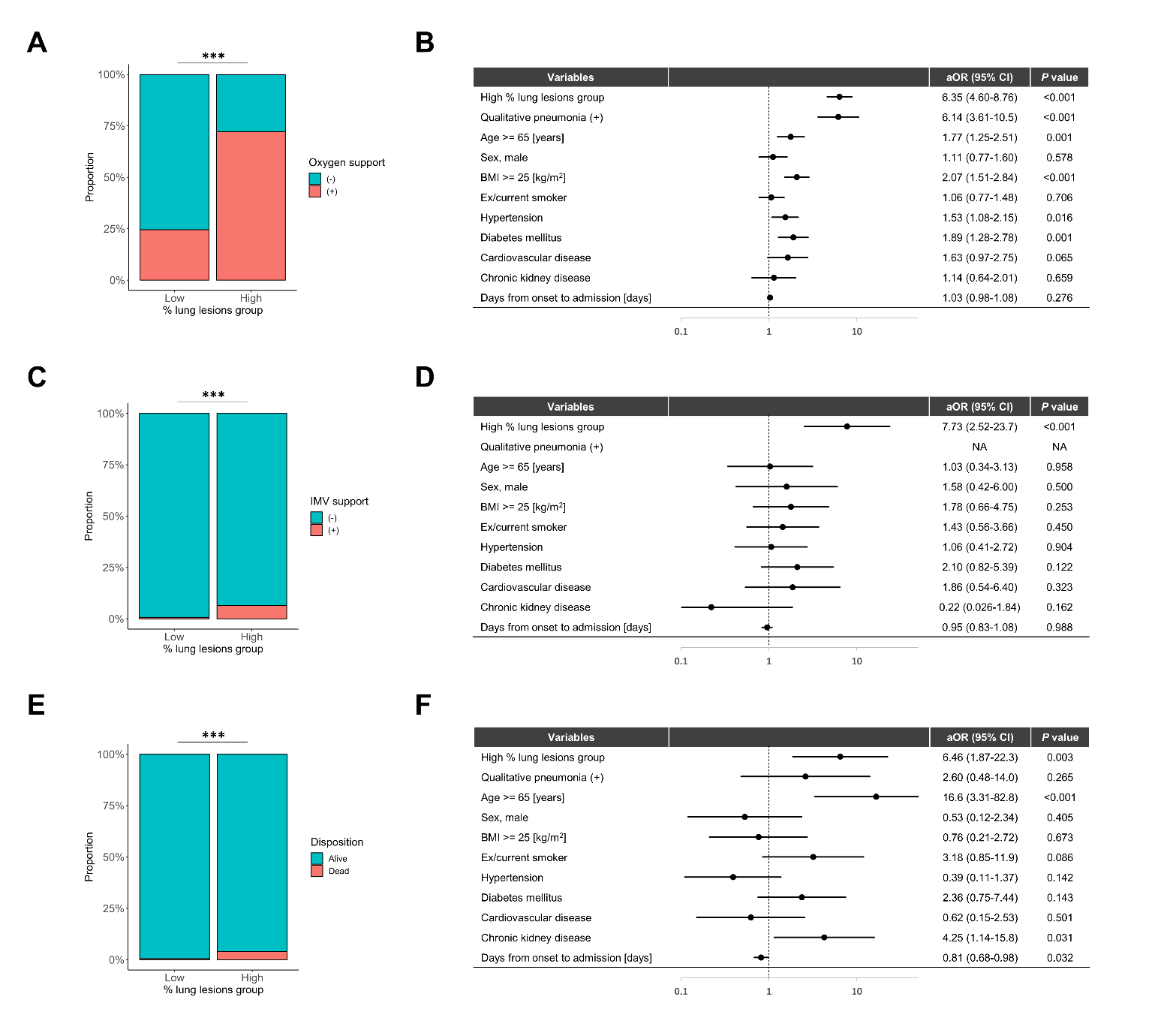


**Figure E5** Relationship between each outcome ([A-B] need for oxygen support during disease course, [C-D] need for IMV support during disease course, [E-F] disposition) and the groups pf pneumonia of high/low % lung lesions on CT images at admission. (A, C, E) The comparison of each outcome between the two groups. (B, D, F) Forest plot showing multivariable logistic regression analysis to evaluate the relationship between each outcome and the high % lung lesions group. aOR; adjusted odds ratio, CI; confidence interval, IMV; invasive mechanical ventilation. ***; *P* < 0.001.

**
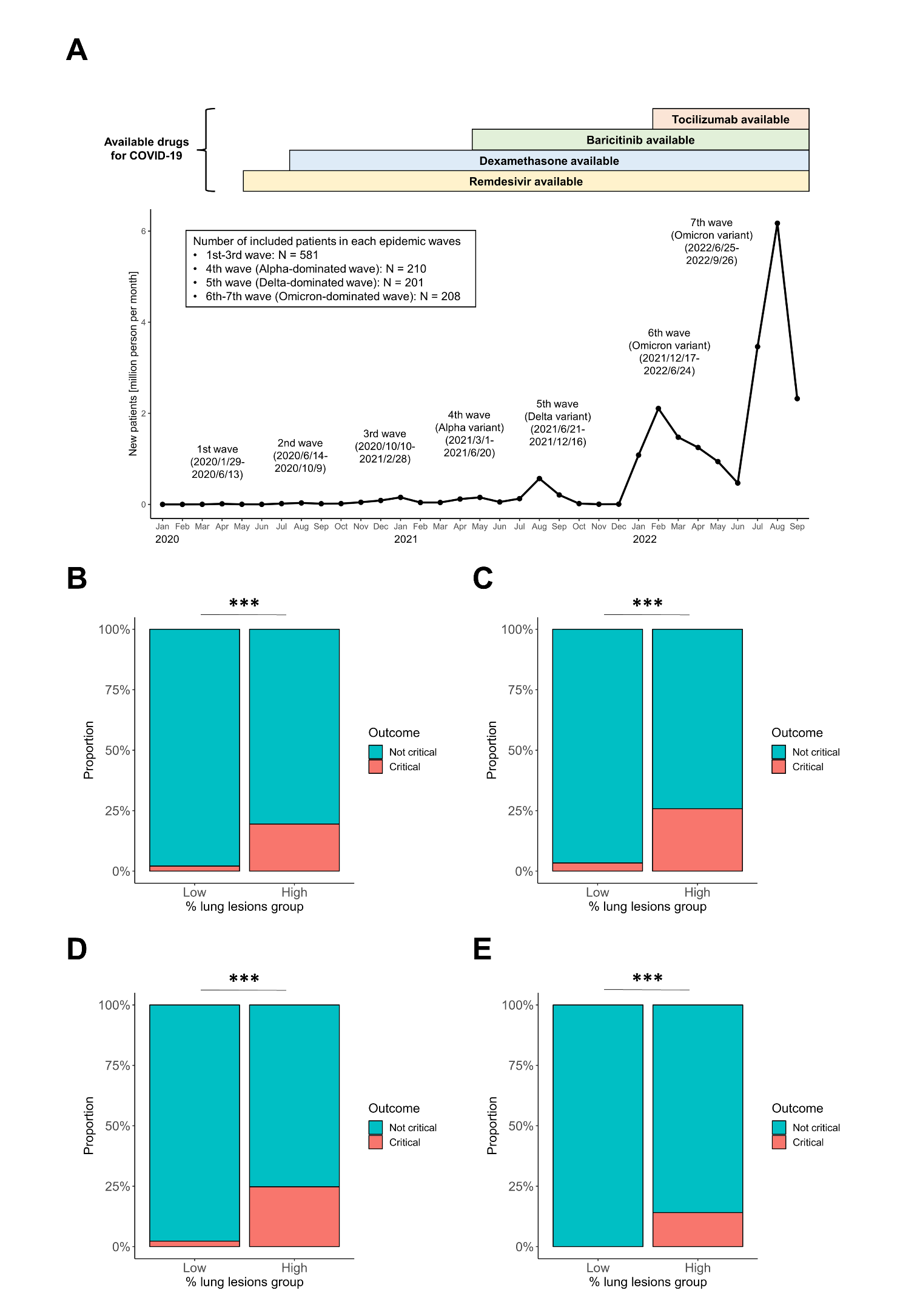
**

**Figure E6** Relationship between critical outcomes and the groups of pneumonia with high/low % lung lesions on CT images at admission, adjusted by epidemic waves in Japan. (A) Number of newly diagnosed COVID-19 patients and seven domestic epidemic waves since January 2021 in Japan. 1st wave; 2020/1/29-2020/6/13, 2nd wave; 2020/6/14-2020/10/9, 3rd wave; 2020-10/10-2021/2/28, 4th wave (Alpha variant-dominated wave); 2021/3/1-2021/6/20, 5th wave (Delta variant-dominated wave); 2021/6/21-2021/12/16, 6th wave (Omicron variant-dominated wave); 2021/12/17-2022/6/24, 7th wave (Omicron variant-dominated wave); 2022/6/25-2022/9/26. (B-E) The comparison of incidence of critical outcomes between the high and low % lung lesions group in each epidemic wave; (B) in 1st-3rd waves, (C) in 4th wave, (D) in 5th wave, and (E) in 6th-7th waves. COVID-19; coronavirus disease 2019. ***; *P* < 0.001.


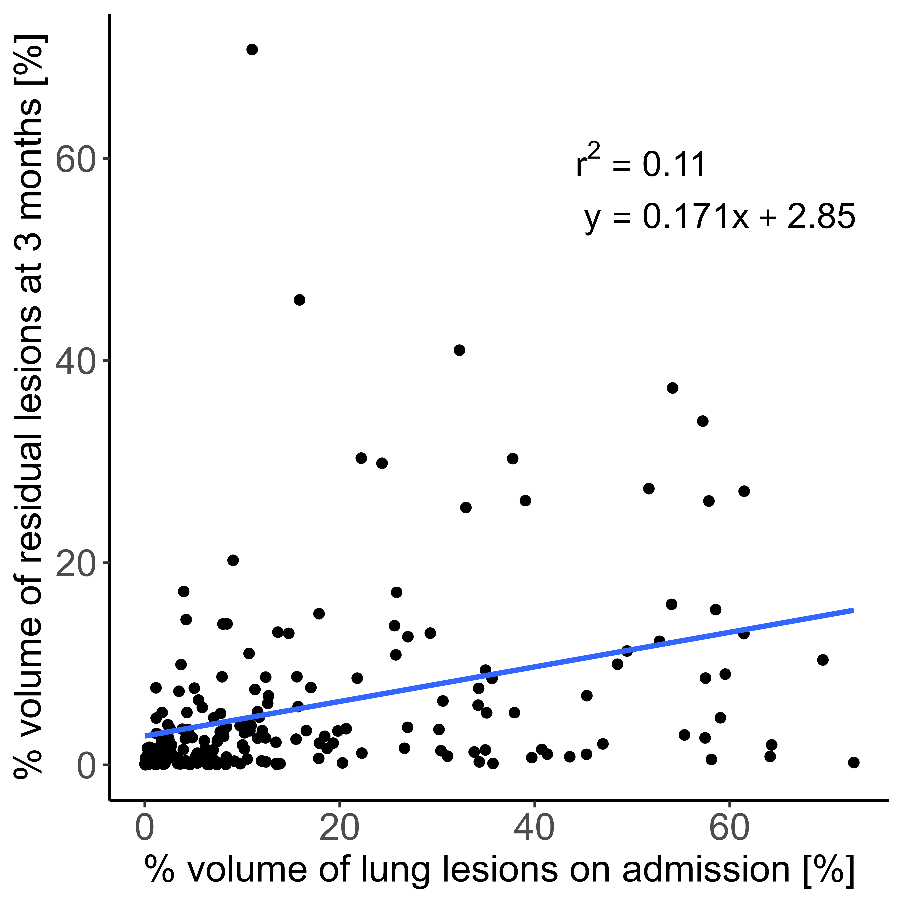


**Figure E7** Scatter diagram and linear regression analysis between % volume of lung lesions on admission and of residual lesions at 3 months, calculated with CT images.

| **Table E1　Comparison of symptoms, laboratory data, and treatments of patients with COVID-19 between groups of pneumonia with high/low percentage of lung lesions on CT images at admission.** | | | | |
| --- | --- | --- | --- | --- |
| **Parameters** |  | **Pneumonia with low**  **% lung lesions group (N = 800)** | **Pneumonia with high**  **% lung lesions group (N = 400)** | ***P* value** |
| Symptoms |  |  |  |  |
| Unconsciousness |  | 11 (1.4) | 13 (3.3) | 0.029 |
| Fever |  | 647 (81.3) | 361 (90.5) | <0.001 |
| Cough |  | 528 (66.0) | 302 (76.1) | <0.001 |
| Sputum |  | 242 (30.5) | 131 (33.0) | 0.377 |
| Sore throat |  | 322 (40.5) | 124 (31.2) | 0.002 |
| Rhinorrhea |  | 192 (24.1) | 76 (19.3) | 0.060 |
| Dysgeusia |  | 162 (20.4) | 87 (21.9) | 0.553 |
| Dysosmia |  | 153 (19.3) | 70 (17.5) | 0.465 |
| Shortness of breath |  | 203 (25.8) | 216 (54.3) | <0.001 |
| Abdominal pain |  | 32 (4.0) | 16 (4.0) | 0.993 |
| Diarrhea |  | 183 (23.0) | 98 (24.6) | 0.538 |
| Nausea or vomiting |  | 95 (12.0) | 73 (18.6) | 0.002 |
| Fatigue |  | 386 (48.6) | 253 (63.6) | <0.001 |
| Laboratory data |  |  |  |  |
| WBC [/µL] |  | 5010 (± 1810) | 6230 (± 3120) | <0.001 |
| Neutrophil [/µL] |  | 3370 (± 1630) | 4850 (± 2680) | <0.001 |
| Lymphocyte [/µL] |  | 1150 (± 510) | 900 (± 430) | <0.001 |
| Albumin [g/dL] |  | 3.94 (± 0.46) | 3.39 (± 0.52) | <0.001 |
| AST [U/L] |  | 33.7 (± 26.4) | 49.4 (± 36.6) | <0.001 |
| ALT [U/L] |  | 33.4 (± 30.7) | 43.6 (± 38.9) | <0.001 |
| BUN [mg/dL] |  | 15.2 (± 9.6) | 19.9 (± 17.5) | <0.001 |
| Creatinine [mg/dL] |  | 1.06 (± 1.56) | 1.56 (± 2.69) | <0.001 |
| LDH [IU/L] |  | 222 (± 69.1) | 342 (± 126) | <0.001 |
| Uric acid [mg/dL] |  | 4.92 (± 1.57) | 4.75 (± 1.81) | 0.107 |
| Ferritin [ng/mL] |  | 409 (± 412) | 903 (± 1140) | <0.001 |
| KL-6 [U/mL] |  | 275 (± 282) | 323 (± 225) | 0.006 |
| HbA1c [%] |  | 6.09 (± 1.03) | 6.48 (± 1.42) | <0.001 |
| D-dimer [µg/mL] |  | 1.22 (± 2.46) | 2.44 (± 6.64) | <0.001 |
| Procalcitonin [ng/mL] |  | 0.15 (± 0.69) | 0.49 (± 1.9) | <0.001 |
| CRP [mg/dL] |  | 2.76 (± 3.84) | 8.35 (± 7.35) | <0.001 |
| Treatments |  |  |  |  |
| Antibiotics |  | 147 (18.4) | 132 (33.0) | <0.001 |
| Favipiravir |  | 156 (19.6) | 68 (17.1) | 0.295 |
| Remdesivir |  | 233 (29.2) | 238 (59.7) | <0.001 |
| Anticoagulant |  | 88 (11.1) | 173 (43.4) | <0.001 |
| Systemic corticosteroid |  | 270 (33.9) | 317 (79.5) | <0.001 |
| Tocilizumab |  | 25 (3.1) | 52 (13.0) | <0.001 |
| Baricitinib |  | 21 (2.6) | 68 (17.0) | <0.001 |
| Data are as N (%) or mean (standard deviation). Abbreviations: ALT, alanine aminotransferase; AST, aspartate aminotransferase; BUN, blood urea nitrogen; COVID-19, coronavirus disease; CRP, C-reactive protein; HbA1c, hemoglobin A 1c; KL-6, Krebs von den Lungen-6; LDH, lactate dehydrogenase; WBC, white blood count. | | | | |

| **Table E2　Comparison of backgrounds, outcomes, and complications of patients with COVID-19 between the 3-month-followed group and non-followed group** | | | | |
| --- | --- | --- | --- | --- |
| **Parameters** |  | **Non-followed group (N = 1002)** | **3-month-followed group (N = 198)** | ***P* value** |
| Age [years] |  | 55.3 (± 17.4) | 56.1 (± 12.8) | 0.516 |
| Sex, male |  | 691 (69.0) | 144 (72.7) | 0.293 |
| BMI [kg/m^2^] |  | 24.8 (± 6.24) | 25.4 (± 4.75) | 0.169 |
| Smoking history |  |  |  | 0.030 |
| Never |  | 537 (55.4) | 89 (46.8) |  |
| Previously or currently |  | 432 (44.6) | 101 (53.2) |  |
| Medical history |  |  |  |  |
| Hypertension |  | 302 (30.2) | 75 (38.1) | 0.030 |
| Diabetes mellitus |  | 176 (17.7) | 43 (21.8) | 0.167 |
| Cardiovascular disease |  | 97 (9.7) | 19 (9.6) | 0.954 |
| Malignancy |  | 103 (10.3) | 9 (4.6) | 0.012 |
| Autoimmune disease |  | 65 (6.5) | 14 (7.2) | 0.735 |
| COPD |  | 25 (2.5) | 9 (4.6) | 0.116 |
| Asthma |  | 70 (7.1) | 16 (8.3) | 0.563 |
| Hyperuricemia |  | 100 (10.1) | 35 (17.7) | 0.002 |
| Chronic liver disease |  | 33 (3.3) | 4 (2.0) | 0.349 |
| Chronic kidney disease |  | 84 (8.5) | 13 (6.6) | 0.395 |
| Severity |  |  |  | <0.001 |
| Critical (= Critical outcomes) |  | 75 (7.5) | 25 (12.6) |  |
| Severe |  | 299 (29.8) | 87 (43.9) |  |
| Mild or asymptomatic |  | 628 (62.7) | 86 (43.4) |  |
| Other outcomes |  |  |  |  |
| Mortality |  | 19 (1.9) | 1 (0.5) | 0.162 |
| IMV support |  | 20 (2.0) | 12 (6.1) | 0.001 |
| ICU admission |  | 228 (22.8) | 30 (15.2) | 0.018 |
| Complications |  |  |  |  |
| Bacterial infection |  | 67 (6.7) | 24 (12.2) | 0.008 |
| Fungal infection |  | 6 (0.6) | 1 (0.5) | 0.872 |
| Heart failure |  | 13 (1.3) | 1 (0.5) | 0.347 |
| Thromboembolism |  | 20 (2.0) | 7 (3.6) | 0.177 |
| Liver dysfunction |  | 392 (40.0) | 120 (61.9) | <0.001 |
| Renal failure |  | 205 (21.0) | 46 (23.6) | 0.414 |
| Data are as N (%) or mean (standard deviation). Abbreviations: BMI, body mass index; COPD, chronic obstructive pulmonary disease; COVID-19, coronavirus disease; ICU, intensive care unit; IMV, invasive mechanical ventilation. | | | | |

| **Table E3　Comparison of backgrounds, symptoms, outcomes, and complications of patients with COVID-19 between groups based on the percentage of residual lesions at 3 months** | | | | |
| --- | --- | --- | --- | --- |
| **Parameters** |  | **Low % residual**  **lesions group (N = 132)** | **High % residual**  **lesions group (N = 66)** | ***P* value** |
| Age [years] |  | 54.4 (± 13.3) | 59.6 (± 11.0) | 0.007 |
| Sex, male |  | 102 (77.3) | 42 (63.6) | 0.042 |
| BMI [kg/m^2^] |  | 25.2 (± 4.71) | 26.0 (± 4.83) | 0.217 |
| Smoking history |  |  |  | 0.766 |
| Never |  | 59 (46.1) | 30 (48.4) |  |
| Previously or currently |  | 69 (53.9) | 32 (51.6) |  |
| Medical history |  |  |  |  |
| Hypertension |  | 41 (31.1) | 34 (52.3) | 0.004 |
| Diabetes mellitus |  | 31 (23.7) | 12 (18.2) | 0.379 |
| Cardiovascular disease |  | 13 (9.9) | 6 (19.1) | 0.865 |
| Malignancy |  | 5 (3.8) | 4 (6.2) | 0.462 |
| Autoimmune disease |  | 8 (6.2) | 6 (9.2) | 0.433 |
| COPD |  | 4 (3.0) | 5 (7.6) | 0.148 |
| Asthma |  | 12 (9.2) | 4 (6.4) | 0.496 |
| Hyperuricemia |  | 23 (17.4) | 12 (18.2) | 0.895 |
| Chronic liver disease |  | 2 (1.5) | 2 (3.1) | 0.455 |
| Chronic kidney disease |  | 6 (4.6) | 7 (10.9) | 0.092 |
| Symptoms |  |  |  |  |
| Unconsciousness |  | 3 (2.3) | 2 (3.1) | 0.736 |
| Fever |  | 121 (92.4) | 60 (90.9) | 0.724 |
| Cough |  | 86 (65.2) | 52 (80.0) | 0.032 |
| Sputum |  | 47 (35.6) | 35 (53.0) | 0.019 |
| Sore throat |  | 44 (33.3) | 26 (39.4) | 0.400 |
| Rhinorrhea |  | 26 (19.7) | 15 (22.7) | 0.620 |
| Dysgeusia |  | 28 (21.4) | 17 (25.8) | 0.489 |
| Dysosmia |  | 28 (21.2) | 15 (22.7) | 0.807 |
| Shortness of breath |  | 57 (44.5) | 34 (51.5) | 0.356 |
| Abdominal pain |  | 7 (5.3) | 3 (4.6) | 0.810 |
| Diarrhea |  | 42 (32.1) | 14 (21.2) | 0.111 |
| Nausea or vomiting |  | 21 (16.0) | 8 (12.3) | 0.490 |
| Fatigue |  | 89 (67.4) | 51 (77.3) | 0.151 |
| Laboratory data |  |  |  |  |
| WBC [/µL] |  | 5110 (± 1930) | 5410 (± 2510) | 0.345 |
| Neutrophil [/µL] |  | 3580 (± 1740) | 4240 (± 2440) | 0.030 |
| Lymphocyte [/µL] |  | 1070 (± 510) | 840 (± 430) | 0.002 |
| Albumin [g/dL] |  | 3.76 (± 0.50) | 3.52 (± 0.49) | 0.002 |
| AST [U/L] |  | 44.0 (± 42.1) | 52.4 (± 37.6) | 0.170 |
| ALT [U/L] |  | 42.4 (± 34.0) | 46.1 (± 43.1) | 0.512 |
| BUN [mg/dL] |  | 15.4 (± 9.38) | 17.3 (± 8.9) | 0.162 |
| Creatinine [mg/dL] |  | 1.11 (± 1.59) | 1.11 (± 1.10) | 0.973 |
| LDH [IU/L] |  | 267 (± 114) | 360 (± 141) | <0.001 |
| Uric acid [mg/dL] |  | 5.00 (± 1.73) | 4.77 (± 1.81) | 0.399 |
| Ferritin [ng/mL] |  | 623 (± 667) | 911 (± 1740) | 0.116 |
| KL-6 [U/mL] |  | 268 (± 103) | 334 (± 158) | 0.002 |
| HbA1c [%] |  | 6.46 (± 1.55) | 6.53 (± 1.33) | 0.765 |
| D-dimer [µg/mL] |  | 0.89 (± 1.05) | 1.55 (± 2.52) | 0.010 |
| Procalcitonin [ng/mL] |  | 0.22 (± 0.79) | 0.18 (± 0.57) | 0.732 |
| CRP [mg/dL] |  | 4.38 (± 5.01) | 7.38 (± 6.19) | <0.001 |
| Severity |  |  |  | <0.001 |
| Critical (= Critical outcomes) |  | 9 (6.8) | 16 (24.2) |  |
| Severe |  | 54 (40.9) | 33 (50.0) |  |
| Mild or asymptomatic |  | 69 (52.3) | 17 (25.8) |  |
| Other outcomes |  |  |  |  |
| Mortality |  | 1 (0.8) | 0 (0.0) | 0.478 |
| IMV support |  | 2 (1.5) | 10 (15.2) | <0.001 |
| ICU admission |  | 14 (10.7) | 16 (24.2) | 0.012 |
| Lengths of hospitalization [days] |  | 12.5 (± 8.5) | 17.4 (± 10.0) | <0.001 |
| Complications |  |  |  |  |
| Bacterial infection |  | 13 (9.9) | 11 (16.9) | 0.159 |
| Fungal infection |  | 0 (0.0) | 1 (1.5) | 0.158 |
| Heart failure |  | 1 (0.8) | 0 (0.0) | 0.473 |
| Thromboembolism |  | 4 (3.1) | 3 (4.6) | 0.608 |
| Liver dysfunction |  | 77 (59.7) | 43 (66.2) | 0.382 |
| Renal failure |  | 24 (18.6) | 22 (33.3) | 0.022 |
| Data are as N (%) or mean (standard deviation). Abbreviations: ALT, alanine aminotransferase; AST, aspartate aminotransferase; BMI, body mass index; BUN, blood urea nitrogen; COPD, chronic obstructive pulmonary disease; COVID-19, coronavirus disease; CRP, C-reactive protein; HbA1c, hemoglobin A 1c; ICU, intensive care unit; IMV, invasive mechanical ventilation; KL-6, Krebs von den Lungen-6; LDH, lactate dehydrogenase; WBC, white blood count. | | | | |

| **Table E4　Univariate logistic regression analysis to evaluate the associated factors with high %residual lesions group** | | |
| --- | --- | --- |
| **Variables** | **OR (95% CI)** | ***P* value** |
| High % pneumonia group on admission | 4.16 (2.21-7.81) | <0.001 |
| Age, >=65 [years] | 2.29 (1.17-4.46) | 0.015 |
| Sex, female | 1.94 (1.02-3.71) | 0.044 |
| BMI, >=25 [kg/m^2^] | 1.31 (0.73-2.38) | 0.366 |
| Smoking history | 0.91 (0.50-1.67) | 0.767 |
| Hypertension | 2.43 (1.32-4.48) | 0.004 |
| Diabetes | 0.72 (0.34-1.51) | 0.381 |
| Cardiovascular disease | 0.92 (0.33-2.53) | 0.865 |
| Autoimmune disease | 1.55 (0.51-4.67) | 0.436 |
| Malignancy | 1.65 (0.43-6.37) | 0.466 |
| COPD | 2.62 (0.68-10.1) | 0.161 |
| Asthma | 0.67 (0.21-2.16) | 0.498 |
| Hyperuricemia | 1.05 (0.49-2.28) | 0.895 |
| Chronic liver disease | 2.10 (0.29-15.2) | 0.464 |
| Chronic kidney disease | 2.58 (0.83-8.02) | 0.102 |
| Abbreviations: BMI, body mass index; CI, confidence interval; COPD, chronic obstructive pulmonary disease; OR, odds ratio. | | |
